# Altered putamen and cerebellum connectivity among different subtypes of Parkinson's disease

**DOI:** 10.1111/cns.13259

**Published:** 2019-11-15

**Authors:** Bo Shen, Yang Pan, Xu Jiang, Zhuang Wu, Jun Zhu, Jingde Dong, Wenbin Zhang, Pingyi Xu, Yakang Dai, Yang Gao, Chaoyong Xiao, Li Zhang

**Affiliations:** ^1^ Department of Geriatrics Affiliated Brain Hospital of Nanjing Medical University Nanjing China; ^2^ Department of Neurosurgery Affiliated Brain Hospital of Nanjing Medical University Nanjing China; ^3^ Department of Neurology The First Affiliated Hospital of Guangzhou Medical University Guangzhou China; ^4^ Suzhou Institute of Biomedical Engineering and Technology Chinese Academy of Sciences Suzhou China; ^5^ Department of Computer Science and Technology Nanjing University Nanjing China; ^6^ Department of Radiology Affiliated Brain Hospital of Nanjing Medical University Nanjing China

**Keywords:** cerebellar, functional connectivity, Parkinson's disease, putamen, tremor

## Abstract

**Objective:**

Impairment of basal ganglia (BG)‐thalamo‐cortical circuit causes various symptoms of Parkinson's disease (PD). We investigated the functional connectivity (FC) patterns of putamen among PD subtypes and healthy control (HC) and explored their clinical significance.

**Methods:**

A total of 16 patients with tremor‐dominant (TD) PD, 23 patients with postural instability and gait difficulty‐dominant (PIGD) PD, and 31 HC that underwent functional magnetic resonance imaging were observed. Voxel‐wise FC analysis was performed by computing correlation between bilateral putamen and other voxels within the brain. Correlation analysis was performed between FC strength and clinical symptoms.

**Results:**

Compared with PIGD group, TD group showed increased FC between left putamen and right cerebellum lobule VI and cerebellum crus I, then we compared the cerebellum FC difference among the three groups. The cerebellum lobule VI FC difference was mainly involved in motor related cortex, and the cerebellum crus I FC difference was related to cognition areas. While compared with HC, TD and PIGD groups both had significant FC difference brain areas correlated with motor and cognition symptoms. The connectively of putamen and right cerebellum lobules VI and I showed positive correlation with tremor and Montreal Cognitive Assessment degree of scores, respectively. The connectivity of putamen and sensorimotor cortex had negative correlation with PIGD scores.

**Conclusions:**

The altered connectivity of BG‐cortical circuit in patients with PD was related to PIGD symptoms. Motor and cognitive impairments declined slower in patients with TD PD, which may be related to increased functional connectivity between putamen and cerebellum.

## 1 INTRODUCTION

Parkinson's disease (PD) is the second common popular progressive neurodegenerative disorder, which always affects people worldwide above the age of 50, which prevalence is expected to 9.3 million by 2030.^1^ At the clinical level, the classic symptoms of PD are resting tremor, muscle rigidity, slowness of movement or bradykinesia and postural instability, patients may have either primary postural instability and gait difficulty‐dominant (PIGD) PD with minimal tremor or tremor‐dominant (TD) PD with minimal rigidity, bradykinesia, and other symptoms.^2^ The TD phenotype has been associated with better cognitive and motor outcomes than with other PD phenotypes.^3^ The PIGD phenotype of PD is associated with a faster rate of cognitive decline and a higher incidence of dementia.^4^ However, the mechanisms of these two phenotypes are unknown.

The pathological hallmark of PD is dopamine neuron loss in the substantia nigra pars compacta, which caused the dysfunction of basal ganglia (BG). Previously, we emphasized the role of BG in modulating cortical function through striatal‐thalamo‐cortical (STC) circuits, the dysfunction of STC circuits usually lead to bradykinesia and rigidity in PD patients.^5^ However, this model cannot explain the resting tremor of PD. Functional MRI (fMRI) has contributed significantly to understand of the pathophysiology underlying neurodegeneration disease, especially to motor manifestations of diseases such as PD. Several fMRI researches suggested altered activity in cerebellar pathways in PD and its progression.^6^ Emerging evidence also suggests the need for incorporating cerebello‐thalamo‐cortical (CTC) circuitry into discussions of motor function in both healthy people and dysfunctional states.^7^ CTC dysfunction is suggested to be involved in tremor in TD PD patients, which could be improved by stimulating thalamic Vim nucleus, as a result of capturing and ameliorating PD tremor.^8^ Therefore, the relationship of BG and cerebellum is unclear in PD progress.

Putamen is the input of BG and receives dopamine from SN. Previous studies showed dopamine difference of putamen among the arranged subtypes of patients with PD. The abnormal activity of putamen leads to the clinical difference of the two subtypes of patients with PD. PET research has found tremor was related to the hypermetabolism of the CTC circuit and putamen.^9^ Another study has shown change in functional connectivity (FC) between the two different PD subtypes with ROI analysis.^10^ They studied the connectivity of BG and motor cortex excluding cerebellum. To our knowledge, no research about the FC of putamen by the voxel‐level analysis within the whole brain has been conducted. In our study, different subtypes of patients with PD were enrolled to investigate putamen FC. Correlation analysis was also used to study whether the altered covariant with motor symptom and cognition level. We hypothesized that patients with TD and PIGD PD must show different connectivity patterns among putamen, cerebellum, and cortical cortex in relation to motor and cognition.

## 2 PARTICIPANTS

Study participants were consecutively recruited from the Department of Geriatrics, Nanjing Brain Hospital from July 2015 to March 2016. Our study enrolled 39 patients with PD and 28 healthy controls (HC). All included patients in the study were satisfied the standard UK Brain Bank Criteria for PD.^11^ Patients were excluded if they had: (a) the history of other neurological or psychiatric diseases, (b) severe head tremor, and (c) cognitive impairment based on PDD Criteria in 2007.^12^ Levodopa equivalent daily dose (LEDD) was calculated using the method described in the previous research.^13^ All participants provided written informed consent, and the study was approved by the Medical Research Ethical Committee of Nanjing Brain Hospital, Nanjing, China.

### 2.1 Assessment of PD motor and cognition symptoms

All patients were tested twice for motor symptoms in two separate days, that is, first assessment was with their regular dopaminergic medication (ON) and another was performed after at least a 12 hours overnight withdrawal from medication (OFF). Clinical assessments including Unified Parkinson's Disease Rating Scale (UPDRS), Hoehn‐Yahr Staging, Mini‐mental State Examination (MMSE), and Montreal Cognitive Assessment (MoCA). Patients with PD were classified into two subgroups, including TD and PIGD subtypes in the off state. When the ratio of the mean tremor score to the mean PIGD score, that is, ≥1.5, was included in the TD subtype PD with PIGD PD when this ratio was ≤1, others were included with mixed subtype PD.^14^ Only TD and PIGD subgroups are enrolled in our analysis.

### 2.2 Magnetic resonance imaging (MRI) data acquisition protocol

All participants underwent a resting‐state fMRI examination by using a 3T system (Siemens, Verio) equipped with an 8‐channel phased‐array head coil in the ON state to minimize head motion. A T1‐weighted images via a volumetric 3D spoiled gradient recall sequence was collected (176 axial slices, repetition time (TR) = 1900 ms, echo time (TE) = 2.48 ms, flip angle (FA) = 9°, matrix = 256 × 256, field of view (FOV) = 250 mm × 250 mm, slice thickness = 1 mm, and gap = 0 mm). Each anatomical run contained 176 image volumes which covered the whole brain with registration and functional localization. Then, a whole brain gradient‐echo echo‐planar imaging sequence (EPI) was collected (TE = 30 ms, TR = 2000 ms, FOV = 240 × 240 mm^2^, slice thickness = 4 mm, slice gap = 0 mm, matrix size = 64 × 64). A total of 240 volumes were acquired from each subject, and the scan duration was 8 minutes and 6 seconds. Participants were instructed to relax, open their eyes to avoid falling asleep, we confirmed the subjects immediately after experiment during fMRI acquisition. Earplugs and cushion was used to minimize scanner noise and head motion, respectively.

### 2.3 Data preprocessing

All resting‐state fMRI data sets were processed using the software of data processing assistant for resting‐state fMRI (DPARSF), which was based on statistical parametric mapping (SPM8).^15^ The first 10 volumes of functional images were discarded for signal equilibrium and participants’ adaptation to scanning noise. Then, the remaining EPI images were preprocessed using following steps: (a) slice time correction, (b) head motion correction by aligning the first image of each session, (c) spatial normalization to a Montreal Neurological Institute (MNI) standard space, (d) smoothing with a Gaussian filter of 8 mm full width at half maximum (FWHM), (e) time series detrending and normalization to a zero mean and a unit variance, and (f) temporary band‐pass filtering (0.01‐0.08 Hz) to remove low‐frequency drifts and physiological high‐frequency noise. No patients had head motions exceeding 3 mm of translation or a rotation of 3°.

### 2.4 Seed region and FC analysis

Bilateral putamen was defined as regions of interest from the WFU_pickatla,^16^ which automatically generated segmented atlas region of interest templates in MNI space. The mean time series of bilateral putamen were extracted. Furthermore, a voxel‐wise FC analysis was performed by computing the temporal correlation between the mean time series of the left or right putamen and the time series of each voxel within the whole brain. The individual correlation coefficients were further transformed to z‐values by using the Fisher r‐to‐z transformation. Therefore, a z‐score map for the entire brain was created for each patient's putamen.

### 2.5 Statistical analysis

PD‐specific clinical characterizations, such as Hoehn and Yahr, MDS‐UPDRS scores, and LEDD, were compared via a two‐sample *t*‐test with subtype as the factor. ACNONA analysis was conducted on the z‐score maps of TD, PIGD, and HCs with age, gender, education level, and gray matter (GM) volumes as covariates, followed by post hoc two‐sample *t*‐tests. Then, between‐group two‐sample *t*‐tests were performed within the mask showing conspicuous differences acquired from ANCOVA analysis. The significance threshold was *P* < .005 with Alphasim correction, with age, gender, education, and GM volumes as covariates.

### 2.6 Behavioral correlations

Behavioral outcomes were selected due to their discriminatory abilities among PD subgroups as determined in their earlier lives. We correlated the putamen FC with motor symptoms in the PD subgroups, such as TD and PIGD scores, and showed that all T maps had a threshold of *P* < .05. Meanwhile, the cluster extent was calculated according to Alphasim correction on the basis of REST software. We also correlated FC with cognition level, and all significant brain areas were among the changed results of previous statistical analysis.

## 3 RESULTS

### 3.1 Clinical and neuropsychological evaluations

Demographic and clinical data from 39 patients with PD and 28 HC are summarized in Table 1. Age, sex, education, and MMSE scores did not differ significantly among the three groups. Tremor scores, PIGD scores, and LEDD differed from the subtype patients with PD (*P* < .05), whereas the total UPDRS scores, UPDRS‐III scores, duration, and H&Y stage did not show any difference. Both PD subgroups showed lower MoCA scores than HCs, and no difference was observed between the two subgroups.

**Table 1:** Demographic and neuropsychological characteristics of all subjects

Group	TD (n = 16)	PIGD (n = 23)	HC (n = 28)	*P* value
Age (year)	58.9 ± 10.4	61.4 ± 8.98	59.12 ± 4.47	.515^a^
Sex (male/female)	6/10	12/11	14/14	.634^b^
Education, year	11.2 ± 4.43	10.8 ± 2.98	11.8 ± 2.41	.556^a^
MMSE	27.5 ± 1.83	27.6 ± 4.54	28.0 ± 1.53	.858^a^
MoCA	25.8 ± 4.09	23.4 ± 5.71	27.0 ± 1.37	.006
Duration (year)	7.16 ± 3.15	9.57 ± 5.00		.097
Tremor scores (off medication)	12.2 ± 5.13	4.26 ± 5.50		<.001
Tremor scores (on medication)	3.69 ± 3.14	1.04 ± 1.64		<.001
PIGD scores (off medication)	3.13 ± 2.28	7.22 ± 4.28		.001
PIGD scores (on medication)	1.75 ± 1.57	3.04 ± 2.08		.042
UPDRS (off medication)	53.6 ± 31.0	65.7 ± 24.1		.202
UPDRS‐III (off medication)	32.6 ± 21.5	37.4 ± 17.3		.464
UPDRS‐III (on medication)	14.9 ± 7.89	18.5 ± 8.50		.183
H&Y stage (off medication)	2.91 ± 0.92	3.46 ± 0.88		.07
Hoehn and Yahr (on medication)	1.86 ± 0.50	2.20 ± 0.54		.065
LEDD	556 ± 211	802 ± 309		.005

Abbreviaitons: LEDD, levodopa equivalent daily dose; MMSE, Mini‐Mental State Examination; MoCA, Montreal Cognitive Assessment; PIGD, postural instability and gait difficulty; TD, tremor‐dominant; UPDRS, unified Parkinson's disease rating scale.

a *P* value was obtained using one‐way analyses of variance.

b *P* value for the gender difference was obtained by chi‐square test, and others were obtained by two‐sample *t*‐test.

### 3.2 ANOVA of three groups

The differences in right putamen FC for brain regions among TD, PIGD, and HC groups were observed in right cerebellar area I, right cerebellum VI, right inferior occipital gyrus, right hippocampus, right inferior temporal gyrus, bilateral supplementary motor area (SMA), right lingual gyrus, right thalamus, left paracentral lobule, left precentral gyrus, and left precentral gyrus (Figure S1, Table S1).

Among patients with TD and PIGD PD and HCs, right putamen FC difference existed in right cerebellums I, IV, V, and VI; right inferior occipital gyrus; right inferior temporal gyrus; right lingual gyrus; right thalamus; and left central lobule (Figure S1, Table S1). The significance threshold of ACNOVA was *P* < .005 with Alphasim correction with voxel‐level *P* < .005 and cluster size of >44 voxels determined by a Monte‐Carlo simulation that resulted in a cluster‐level significance threshold of *P* < .005.

### 3.3 TD vs patients with PIGD PD

**Figure 1:**
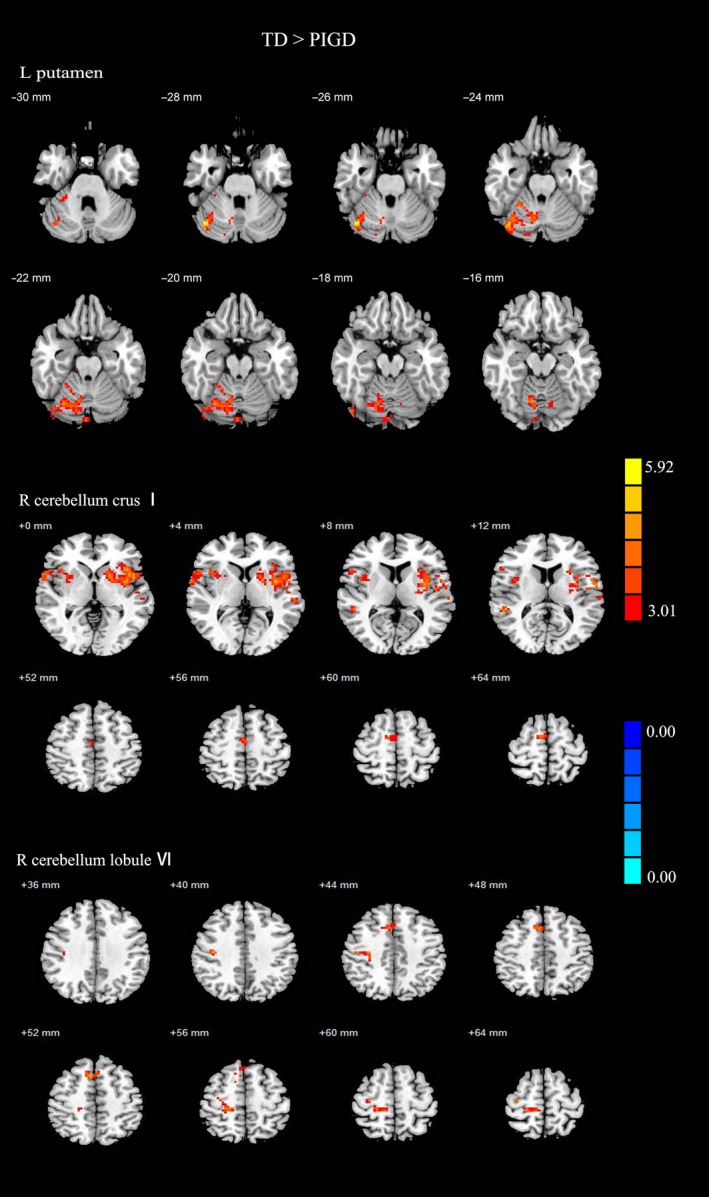
Results of two‐sample *t*‐test between TD PD patients and PIGD PD patients. All results are in MNI space, red color represents the increased FC of left putamen, while the blue color represents the decreased FC, thresholded at *P* < .005, corrected using alphasim in REST

Compared with PIGD group, TD group have enhanced FC between left putamen and right cerebellum lobule VI (the peak MNI coordinates were 13, −65, and −14; the T value was 4.24; and the voxel size was 124) and cerebellum crus I (the peak MNI coordinates were 39, −69, and −27; the T value was 5.51; and the voxel size was 47). No difference in FC was observed between the two groups in terms of the right putamen. Then, we select the changed areas as ROI to compared the FC within the whole brain between the different subtypes PD patients, the centers of ROI were the peak MNI coordinates and radius for each ROI was 4 mm, followed by ACNONA analysis among the three groups and post hoc two‐sample *t*‐tests. Compared with PIGD group, TD groups showed increased FC between right cerebellum crus I and right opercular part of inferior frontal gyrus, left insula, left putamen, left rolandic operculum, right SMA, what's more, increase FC between right cerebellum lobule VI and right precentral gyrus, right postcentral gyrus, right SMA were also observed in TD groups (Figure 1, Table S2).

### 3.4 PIGD vs HC subjects

**Figure 2:**
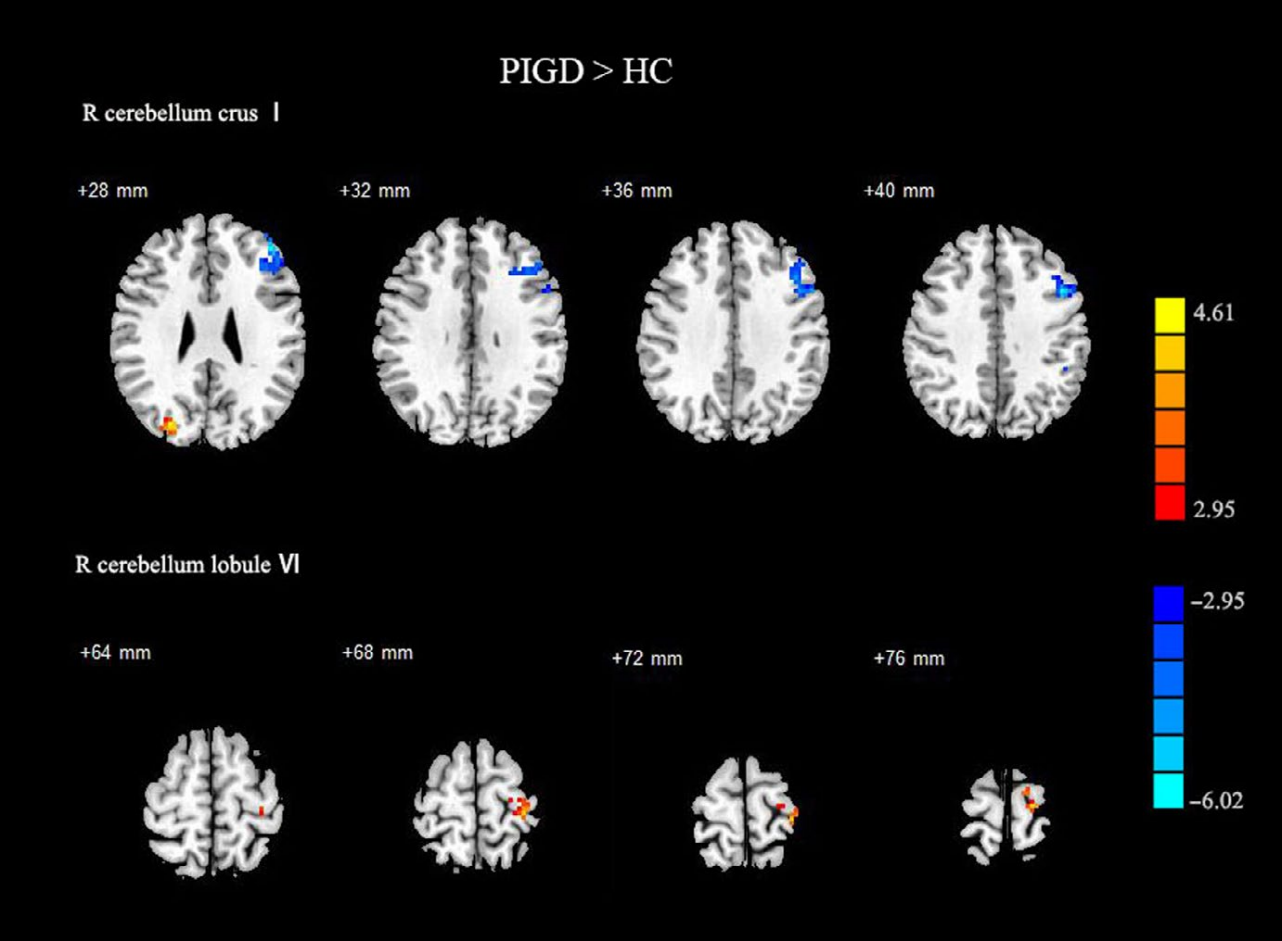
Results of two‐sample *t*‐test between PIGD PD patients and HC subjects. All results are in MNI space, red color represents the increased FC of left putamen, while the blue color represents the decreased FC, thresholded at *P* < .005, corrected using alphasim in REST

While compared with HC, PIGD group showed decreased FC between right cerebellum crus I and left middle frontal gyrus, left inferior parietal lobule, and inferior frontal gyrus, increased FC patterns of right cerebellum gyrus VI were located in left precentral gyrus and left postcentral gyrus. Insignificant difference in the bilateral putamen FC existed between the PIGD and healthy groups (Figure 2, Table S3).

### 3.5 TD vs HC subjects

**Figure 3:**
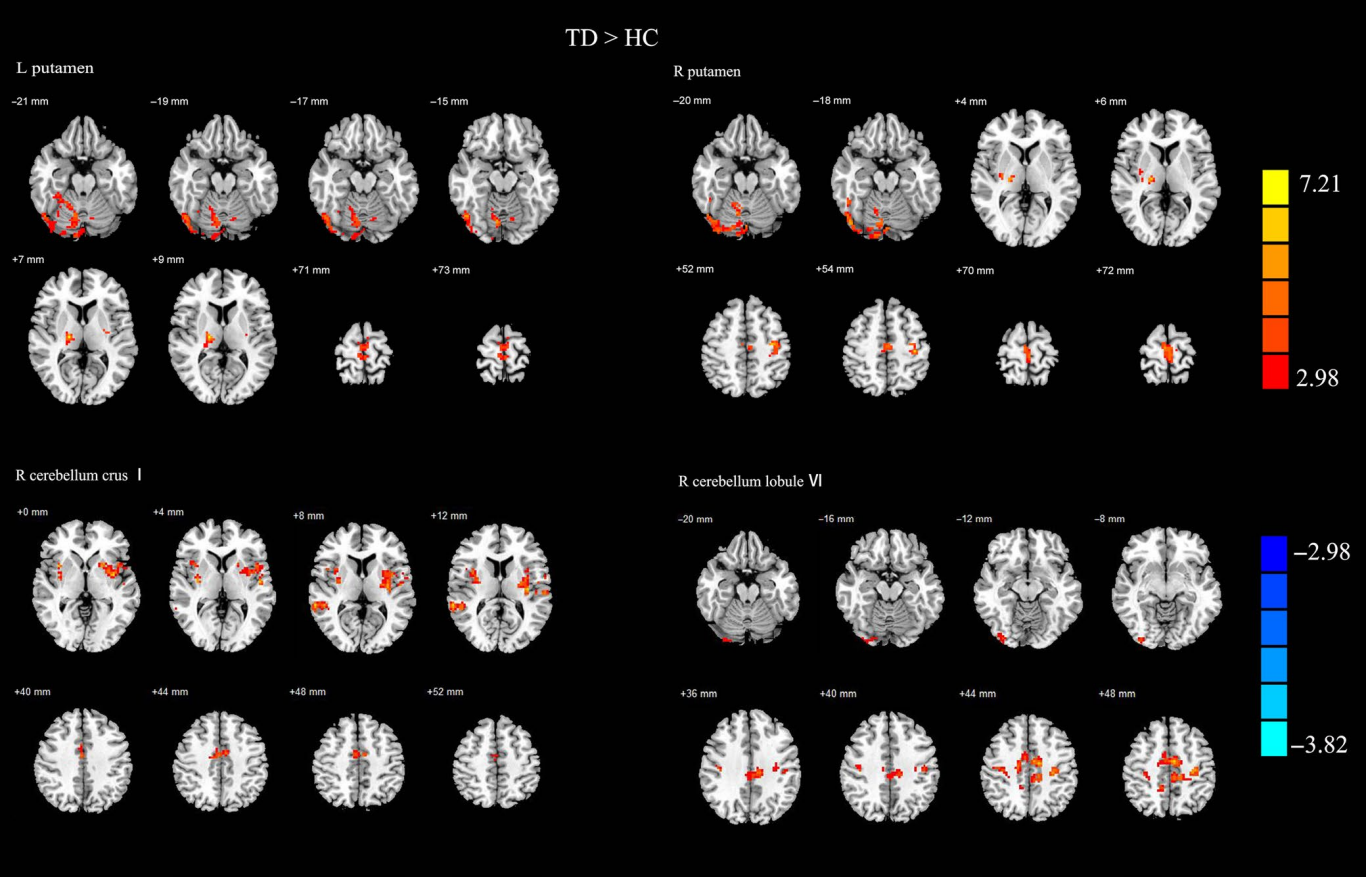
Results of two‐sample *t*‐test between TD PD patients and HC subjects. All results are in MNI space, red color represents the increased FC of left putamen, while the blue color represents the decreased FC, thresholded at *P* < .005, corrected using alphasim in REST

In comparison with that in the HC group, patients with TD PD showed increased FC between left putamen and bilateral cerebellum crus I, right cerebellum lobule IV, V and VI, right thalamus, left paracentral lobule, right inferior occipital lobule, cerebellar vermis, and left SMA. Patients with TD PD also showed increased connectivity between right putamen and right cerebellum crus I, right cerebellum lobule VI, right thalamus, bilateral paracentral lobule, right inferior occipital lobule, right inferior temporal gyrus, bilateral SMA, and bilateral precentral lobule (Figure 3, Table S4). What's more, right cerebellum crus I seed had higher FC with the bilateral insula, putamen, superior temporal gyrus, and right middle cingulate gyrus; higher right cerebellum gyrus VI FC pattern was located in bilateral precentral gyrus, postcentral gyrus, SMA, paracentral lobule, middle cingulate gyrus, and left precuneus.

### 3.6 Correlation analysis

**Figure 4:**
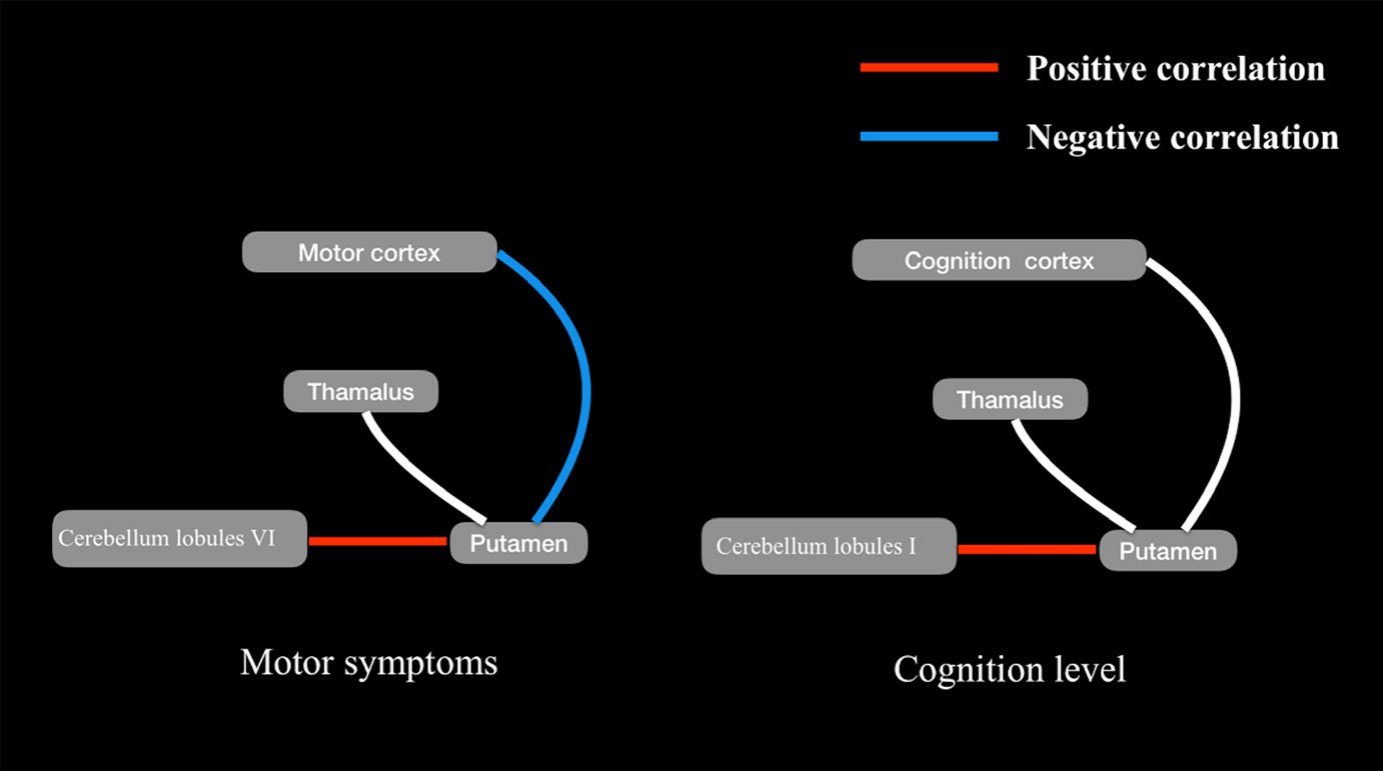
Correlation Analysis of FC and clinical symptom. All T maps had a threshold of *P* < .05, and cluster extent >20, according to Alphasim correction. A: The red line represents the positive correlation of UPDRS III, and blue line represents the negative correlation of UPDRS III. B: The red line represents the positive correlation of MoCA scores

FC strength between the left putamen and right cerebellum lobule VI (the peak MNI coordinates were 12, −76, and −20; and the voxel size was 22) showed positive relationship with tremor scores in TD group, and the FC strength between right putamen and left sensorimotor cortex (the peak MNI coordinates were 30, −24, and 54; and the voxel size was 17) showed negative relationship with PIGD scores (the peak MNI coordinates were 33, −66, and −24; and the voxel size was 17). The connection of putamen and cerebellum crus I had a positive relationship with MoCA scores (Figure 4).

## 4 DISCUSSION

In this study, FC patterns of putamen in PIGD, TD, and control groups were assessed to detect group differences. We found that the TD subgroup (compared to the PIGD subgroup) had higher FC between the left putamen and the right cerebellum, the alterations which is strengthened by the relations of the motor and cognitive domains. In the following paragraphs, we will discuss the neuroimaging patterns that were within either PIGD or TD subgroup.

Compared with PIGD PD patients, TD subgroup had higher FC between left putamen and right cerebellum lobule VI, followed by cerebellum lobule VI FC analysis mainly involved with motor related brain areas, such as right precentral gyrus, right postcentral gyrus, and right SMA. Previous research found that the internal globus pallidus (GPi) drives tremulous activity in a CTC motor loop through the motor cortex, it is a well‐accepted model to explain the tremor symptoms.^17^ What's more, the subthalamic nucleus of BG has a substantial disynaptic projection to cerebellar cortex. This pathway provided a means for signals from BG to influence cerebellar function.^18^ In our study, PD patients showed increased FC between putamen and cerebellum, which indicates strong connection between BG and cerebellum. Task fMRI studies have shown that cerebellar activity was increased when patients with PD completed the autonomic action.^19^ Resting‐state fMRI studies have also shown that patients with PD have enhanced degree of cerebellar activity.^20^ In addition, in the completion of the action, the effective connection between cerebellum and motor cortex was increased.^21^ Gao et al also used electromyogram and fMRI to record the relationship between muscle activity and brain function. At the beginning of tremor, patients with PD showed BG activation. With continuous tremor, cerebellar activation was increased, and the connection among medial GPi, putamen, and cerebellum function was increased.^22^ Otherwise, the connectively of left putamen and right cerebellum lobule VI showed positive relationship with tremor scores in TD group, and the FC strength between right putamen and left sensorimotor cortex showed negative relationship with PIGD scores. These results suggested that Parkinson's tremor may be the result of STC loops injury and CTC loops compensation.^23^ The increased connectivity of putamen and cerebellum in TD PD may indicate the direct effect of putamen on cerebellum, which is likely to be related to a slower disease progression.

When compared with PIGD PD patients, TD subgroup had higher FC between left putamen and right cerebellum crus I, followed by cerebellum crus I FC analysis mainly involved with cognition related brain areas, such as inferior frontal gyrus and left Insula. Previous study had showed hyperactivity in the opercular part of right inferior frontal lobe in the PD patients with mild cognitive impairment, when compared to both HC and PD patients with normal cognitive level.^24^ Inferior frontal lobe has been broadly suggested to play an important role in executive control function in many task fMRI researches.^25^ Insula, combined with default mode network, has also been confirmed on cognitive processing among aging individuals and individuals with neurodegenerative disorders in recent fMRI studies.^26^ Rolandic opercular and SMA are the two brain areas associated with motor symptoms.^27^ Conjunction analysis indicated that individuals with PD had activation in bilateral cerebellum, SMA, ipsilateral precentral gyrus, and postcentral gyrus during both self‐ and cue‐initiated movement. PD patients exhibited enhanced brain activity in the cerebellum crus I and II during self‐initiated movement.^28^ In conclusion, the cerebellum crus I and II are mainly involved with cognition and also as a part of execution control, combined with cerebellum VI, modulate the movement control in the Parkinson's disease. In our study, increased FC between putamen and cerebellum crus I was correlated to MoCA scores in patients with TD PD, because they always have slow progressive cognition decline.

In comparison with that in the HC group, TD patients had all increased FC pattern related to motor and cognition. Putamen, thalamus, and motor cortex consist of the classic STC circuits, which activity increased correlated with motor symptoms of PD,^29^ and DBS could release this activity, improved the movement control.^30^ What's more, many cognition related areas were also found in the cerebellar connectivity. PIGD PD patients also showed increased FC between cerebellum lobule VI and sensorimotor cortex, but in contrast to TD patients, decreased FC between cerebellum crus I and cognition related areas, such as middle frontal gyrus, inferior parietal gyrus, inferior frontal gyrus, and triangular part.^31^, ^32^ We guess that this was the damage phenomenon related to higher speed of cognition decline.^33^

Compared with other articles, we enrolled the patients on medication. Previous study has found the beneficial effects of levodopa on Parkinson's disease, bradykinesia, are associated with alteration of the STC and STN‐cortical motor pathways.^34^ Our research also proved that the functional connectivity also relates to motor symptoms on medication.^35^

In summary, the BG‐cortical loops of patients with PD were related to PIGD symptoms, and connectivity between putamen and cerebellum was correlated to TD scores and MoCA scores in TD patients, respectively. Compared with patients with PIGD PD, motor symptoms and cognitive levels decreased slowly in patients with TD PD. This result may be related to the increased FC between BG and cerebellum.

## CONFLICT OF INTEREST

All authors report no conflict of interest.
