# Supplementary figures and images for: Altered putamen and cerebellum connectivity among different subtypes of Parkinson's disease
Source: CNS Neurosci Ther. 2019 Nov 15;26(2):207–14. doi: 10.1111/cns.13259 (PMC6978269; doi:10.1111/cns.13259)

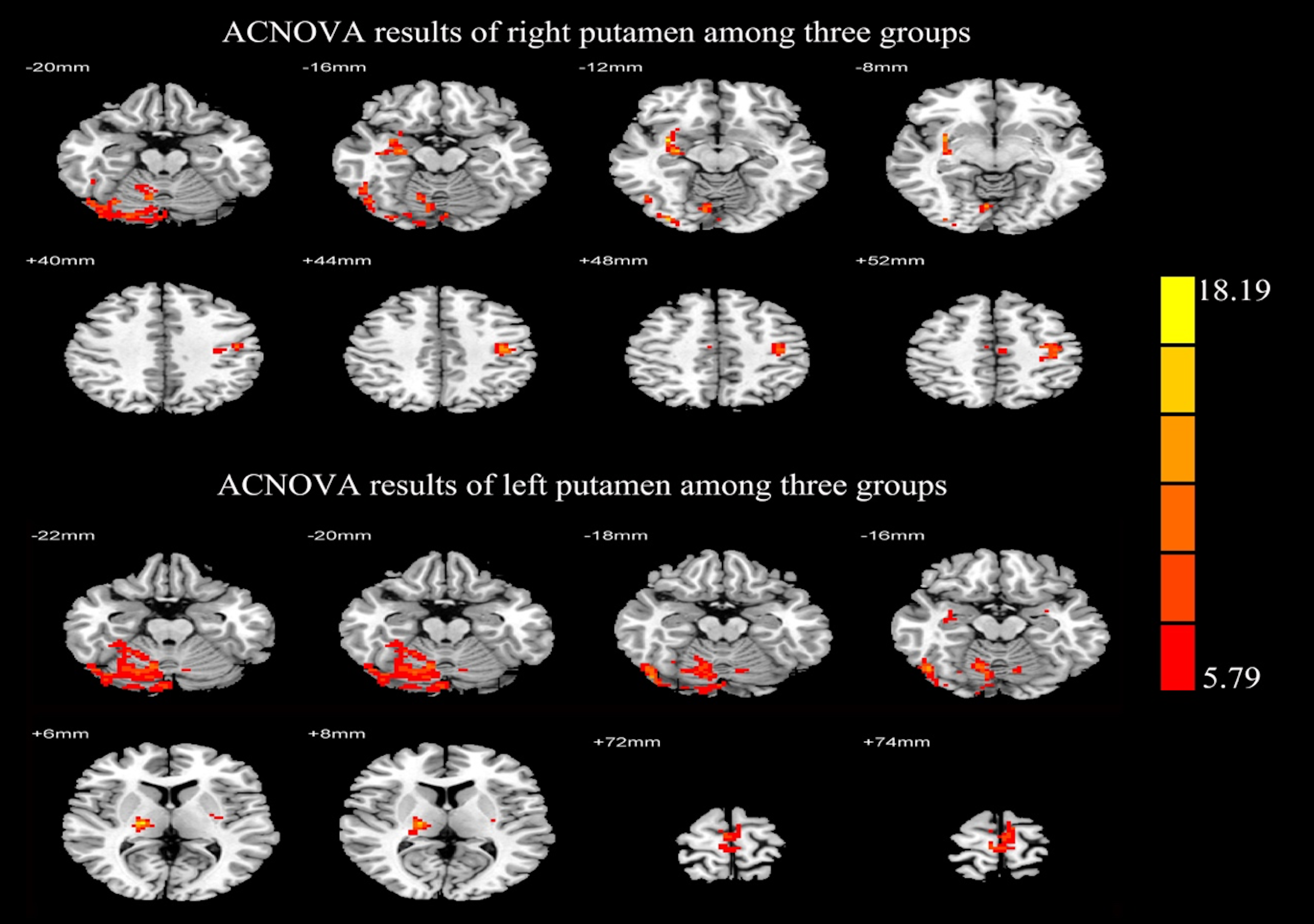

Supplement: Supplementary file 1 [file CNS-26-207-s001.png]
