# Supplementary material for: Altered putamen and cerebellum connectivity among different subtypes of Parkinson's disease
Source: CNS Neurosci Ther. 2019 Nov 15;26(2):207–14. doi: 10.1111/cns.13259 (PMC6978269; doi:10.1111/cns.13259)
Supplement: Supplementary file 2 [file CNS-26-207-s002.docx]

**Table. S1**：ACNOVA results of putamen FC among TD, PIGD, and HC

| Seed | ROI | MNI coordinates | | | Voxel sizes | F values |
| --- | --- | --- | --- | --- | --- | --- |
|  |  | x | y | z |  |  |
| Left putamen | R cerebellum lobule VI | 11 | –65 | -24 | 215 | 13.86 |
|  | R cerebellum crus I | 39 | -69 | -27 | 87 | 20.02 |
|  | R inferior occipital gyrus | 47 | –66 | -12 | 27 | 15.45 |
|  | R lingual gyrus | 9 | –79 | -12 | 23 | 9.96 |
|  | R inferior temporal gyrus | 52 | -63 | -19 | 23 | 6.56 |
|  | R thalamus | 18 | –18 | 6 | 58 | 20.67 |
|  | L paracentral lobule | –6 | -18 | 75 | 34 | 9.77 |
| Right putamen | R cerebellum lobule VI | 11 | –62 | -22 | 70 | 10.14 |
|  | R cerebellum crus I | 39 | -72 | -24 | 111 | 11.72 |
|  | R hippocampus | 27 | –11 | -11 | 22 | 13.79 |
|  | R lingual gyrus | 6 | -73 | -9 | 28 | 7.85 |
|  | R inferior occipital gyrus | 33 | –90 | -12 | 25 | 13.34 |
|  | R inferior temporal gyrus | 53 | -62 | -17 | 23 | 6.65 |
|  | R thalamus | 18 | –18 | 6 | 43 | 14.09 |
|  | L paracentral lobule | –6 | -18 | 75 | 65 | 10.44 |
|  | L precentral gyrus | -37 | -11 | 51 | 37 | 7.86 |
|  | L procentral gyrus | -36 | -24 | 54 | 84 | 13.01 |
|  | R SMA | 3 | -18 | 72 | 31 | 11.22 |
|  | L SMA | -4 | -18 | 53 | 22 | 7.19 |

Spatial distribution of significant voxels with respect to their locations according to the automated anatomical labeling AAL template, results are in MNI space. ROI: Region Of Interest.
