# Supplementary material for: Altered putamen and cerebellum connectivity among different subtypes of Parkinson's disease
Source: CNS Neurosci Ther. 2019 Nov 15;26(2):207–14. doi: 10.1111/cns.13259 (PMC6978269; doi:10.1111/cns.13259)
Supplement: Supplementary file 3 [file CNS-26-207-s003.docx]

Table 4. Functional connectivity differences between TD patients and PIGD patients.

| Seed | ROI | Peak coordinates | | | Voxel size | *T* value |
| --- | --- | --- | --- | --- | --- | --- |
|  |  | x | y | z |  |  |
| L putamen | R cerebellum lobule VI | 13 | -65 | -14 | 124 | 4.24 |
|  | R cerebellum crus I | 39 | -69 | -27 | 47 | 5.51 |
| R cerebellum crus I | R inferior frontal gyrus, opercular part | 53 | 15 | 9 | 30 | 3.81 |
|  | L insula | -39 | 18 | -3 | 162 | 5.92 |
|  | L putamen | -19 | 10 | -4 | 91 | 4.62 |
|  | L inferior frontal gyrus, opercular part | -56 | 7 | 17 | 43 | 4.12 |
|  | L rolandic operculum | -58 | -1 | 12 | 39 | 4.24 |
|  | R SMA | 6 | 0 | 45 | 49 | 4.31 |
| R cerebellum lobule VI | R precentral gyrus | 45 | -17 | 44 | 34 | 4.27 |
|  | R postcentral gyrus | 39 | -17 | 40 | 28 | 4.11 |
|  | R SMA | 9 | 18 | 51 | 47 | 4.36 |

Spatial distribution of significant voxels with respect to their locations according to the automated anatomical labeling AAL template, results are in MNI space. ROI: Region of interest.
