# Supplementary material for: Altered putamen and cerebellum connectivity among different subtypes of Parkinson's disease
Source: CNS Neurosci Ther. 2019 Nov 15;26(2):207–14. doi: 10.1111/cns.13259 (PMC6978269; doi:10.1111/cns.13259)
Supplement: Supplementary file 4 [file CNS-26-207-s004.docx]

Table 3. Functional connectivity differences between PIGD patients and HCs

| Seed | ROI | MNI coordinate | | | Voxel sizes | P value |
| --- | --- | --- | --- | --- | --- | --- |
|  |  | X | Y | Z |  |  |
| R cerebellum crus I | L middle frontal gyrus | -48 | 12 | 42 | 116 | -6.01 |
|  | L inferior frontal gyrus, triangular part | -43 | 27 | 32 | 31 | -3.94 |
|  | L inferior parietal gyrus | -48 | -48 | 51 | 106 | -4.67 |
| R cerebellum lobule VI | L precentral gyrus | -15 | 12 | 78 | 22 | 4.69 |
|  | L postcentral gyrus | -34 | -29 | 68 | 16 | 4.19 |

Spatial distribution of significant voxels with respect to their locations according to the automated anatomical labeling AAL template, results are in MNI space. ROI: Region of interest.
