# Supplementary material for: Altered putamen and cerebellum connectivity among different subtypes of Parkinson's disease
Source: CNS Neurosci Ther. 2019 Nov 15;26(2):207–14. doi: 10.1111/cns.13259 (PMC6978269; doi:10.1111/cns.13259)
Supplement: Supplementary file 5 [file CNS-26-207-s005.docx]

Table 2. Functional connectivity differences between TD patients and HCs

| Seed | ROI | | Peak coordinates | | | Voxel size | *T* value |
| --- | --- | --- | --- | --- | --- | --- | --- |
|  |  |  | x | y | z |  |  |
| L putamen | | R cerebellum lobule VI | 9 | –75 | -15 | 116 | 5.12 |
|  | | R cerebellum crus I | 39 | –70 | -25 | 44 | 4.42 |
|  | | R thalamus | 18 | -17 | 5 | 51 | 6.46 |
|  | | L paracentral lobule | -6 | -18 | 75 | 34 | 4.72 |
| R putamen | | R cerebellum lobule VI | 9 | –76 | -16 | 41 | 4.16 |
|  | | R cerebellum crus I | 39 | –73 | -24 | 86 | 4.01 |
|  | | R thalamus | 18 | -17 | 10 | 38 | 5.57 |
|  | | R precentral lobule | -39 | -18 | 46 | 75 | 5.13 |
|  | | L precentral lobule | -33 | -14 | 50 | 31 | 4.94 |
|  | | L paracentral lobule | -3 | -20 | 74 | 65 | 4.54 |
|  | | R SMA | 3 | -17 | 72 | 30 | 4.48 |
| R cerebellum crus I | | L insula | -35 | -17 | 12 | 92 | 4.75 |
|  | | L putamen | -23 | 10 | -4 | 83 | 4.86 |
|  | | L superior temporal gyrus | -60 | -6 | -6 | 48 | 4.89 |
|  | | R insula | 33 | -10 | 4 | 53 | 4.26 |
|  | | R putamen | 29 | -5 | 12 | 39 | 3.96 |
|  | | R superior temporal gyrus | 63 | -39 | 12 | 44 | 4.86 |
|  | | R median cingulate and paracingulate gyrus | 3 | -9 | 42 | 34 | 4.25 |
| R cerebellum lobule VI | | L paracentral lobule | -6 | -24 | 57 | 155 | 7.21 |
|  | | L postcentral gyrus | -30 | -34 | 64 | 139 | 4.75 |
|  | | R SMA | 3 | -26 | 56 | 120 | 5.50 |
|  | | R precentral gyrus | 21 | -29 | 64 | 110 | 5.86 |
|  | | L median cingulate and paracingulate gyrus | -5 | -8 | 48 | 105 | 6.21 |
|  | | R postcentral gyrus | 16 | -32 | 64 | 139 | 6.56 |
|  | | L SMA | -5 | -8 | 52 | 91 | 5.28 |
|  | | L precentral gyrus | -30 | -29 | 64 | 90 | 5.81 |
|  | | R paracentral lobule | 13 | -32 | 60 | 69 | 6.24 |
|  | | L Precuneus gyrus | -9 | -40 | 68 | 56 | 4.23 |
|  | | R median cingulate and paracingulate gyrus | 6 | -3 | 44 | 51 | 3.78 |

Spatial distribution of significant voxels with respect to their locations according to the automated anatomical labeling AAL template, results are in MNI space. ROI: Region of interest.
