# Supplementary material for: Altered putamen and cerebellum connectivity among different subtypes of Parkinson's disease
Source: CNS Neurosci Ther. 2019 Nov 15;26(2):207–14. doi: 10.1111/cns.13259 (PMC6978269; doi:10.1111/cns.13259)
Supplement: Supplementary file 6 [file CNS-26-207-s006.docx]

**Supplement materials**

Fig.S1. ACNOVA results of bilateral putamen among PD subgroups and HC subjects.

Fig.S1 ACNOVA results of bilateral putamen among PD subgroups and HC. results are in MNI space, red color represents different brain area of FC among the three groups.

Table. S1：ACNOVA results of putamen FC among TD, PIGD, and HCs

Spatial distribution of significant voxels with respect to their locations according to the automated anatomical labeling AAL template, results are in MNI space. ROI: Region Of Interest.

Table S2. Functional connectivity differences between TD PD patients and PIGD PD patients

Table S2. Spatial distribution of significant voxels with respect to their locations according to the automated anatomical labeling AAL template, results are in MNI space. ROI: Region Of Interest.

Table S3. Functional connectivity differences between PIGD PD patients and TD PD patients

Table S3. Spatial distribution of significant voxels with respect to their locations according to the automated anatomical labeling AAL template, results are in MNI space. ROI: Region Of Interest.

Table S4. Functional connectivity differences between TD PD patients and PIGD PD patients

Table S4. Spatial distribution of significant voxels with respect to their locations according to the automated anatomical labeling AAL template, results are in MNI space. ROI: Region Of Interest.
